# Extracellular Vesicle lincRNA-p21 Expression in Tumor-Draining Pulmonary Vein Defines Prognosis in NSCLC and Modulates Endothelial Cell Behavior

**DOI:** 10.3390/cancers12030734

**Published:** 2020-03-20

**Authors:** Joan J. Castellano, Ramon M. Marrades, Laureano Molins, Nuria Viñolas, Jorge Moises, Jordi Canals, Bing Han, Yan Li, Daniel Martinez, Mariano Monzó, Alfons Navarro

**Affiliations:** 1 Molecular Oncology and Embryology Laboratory, Human Anatomy Unit, School of Medicine, University of Barcelona, IDIBAPS, 08036 Barcelona, Spain; joan.castellano@ub.edu (J.J.C.); canals.serrat@ub.edu (J.C.); bhanhanx7@ub.edu (B.H.); yanli@clinic.cat (Y.L.); mmonzo@ub.edu (M.M.); 2 Thoracic Oncology Unit, Hospital Clinic, 08036 Barcelona, Spain; marrades@clinic.cat (R.M.M.); lmolins@clinic.cat (L.M.); nvinolas@clinic.cat (N.V.); jrmoises@clinic.cat (J.M.); dmartin1@clinic.cat (D.M.); 3 Department of Pneumology, Institut Clínic Respiratori (ICR), Hospital Clínic de Barcelona, University of Barcelona, IDIBAPS, CIBER Enfermedades Respiratorias (CIBERES), 08036 Barcelona, Spain; 4 Department of Thoracic Surgery, Hospital Clínic de Barcelona, University of Barcelona, 08036 Barcelona, Spain; 5 Department of Medical Oncology, Institut Clínic de Malaties Hemato-Oncològiques (ICMHO), Hospital Clínic de Barcelona, University of Barcelona, IDIBAPS, 08036 Barcelona, Spain; 6 Department of Pathology, Hospital Clínic de Barcelona, University of Barcelona, 08036 Barcelona, Spain

**Keywords:** lincRNA-p21, lncRNAs, NSCLC, exosomes, extracellular vesicles, tumor-draining vein, angiogenesis

## Abstract

Hypoxia-induced upregulation of lincRNA-p21 in tumor tissue was previously shown by our group to be related to poor prognosis in resected non-small cell lung cancer (NSCLC) patients. In the present study, we have evaluated the presence of lincRNA-p21 in extracellular vesicles (EVs) from NSCLC patients and assessed its potential as a prognostic biomarker. High EV lincRNA-p21 levels in blood from the tumor-draining vein were associated with shorter time to relapse and shorter overall survival. Moreover, the multivariate analysis identified high lincRNA-p21 levels as an independent prognostic marker. In addition, lincRNA-p21 was overexpressed in H23 and HCC44 NSCLC cell lines and their derived EVs under hypoxic conditions. Functional assays using human umbilical vein endothelial cells (HUVECs) showed that tumor-derived EVs enriched in lincRNA-p21 affected endothelial cells by promoting tube formation and enhancing tumor cell adhesion to endothelial cells. Additionally, the analysis of selected EV microRNAs related to angiogenesis and metastasis showed that the microRNAs correlated with EV lincRNA-p21 levels in both patients and cell lines. Finally, EV co-culture with HUVEC cells increased the expression of microRNAs and genes related to endothelial cell activation. In conclusion, EV lincRNA-p21 acts as a novel prognosis marker in resected NSCLC patients, promoting angiogenesis and metastasis.

## 1. Introduction

Lung cancer is the most common cancer, accounting for 2.1 million new cases annually, and is the leading cause of death from cancer, with 1.8 million deaths per year [1]. In early-stage non-small-cell lung cancer (NSCLC) [2], potentially curative surgery is the gold standard of treatment, combined or not with adjuvant chemotherapy [3]. However, up to 40% of resected patients with stage I, II, or IIIA will relapse in the first five years after surgery [4], indicating the urgent need to develop new biomarkers to identify patients at high risk of relapse to improve their survival rates after surgery. The analysis of blood, the so-called “liquid biopsy”, can be used to identify biomarkers, including the study of circulating extracellular vesicles (EVs), such as exosomes [5,6]. Most papers define exosomes as small EVs ranging from 30 to 150 nm in diameter [7]. However, according to MISEV2018 [8], when exosomes are only defined according to physical characteristics like size, they should be referred to as small EVs. Exosome secretion is finely regulated by tumor cells and they are key players in cell-to-cell communication during tumor malignization [9]. Our group has previously shown that primary tumor-related EVs can be obtained from the tumor-draining pulmonary vein in resected patients and that the study of their physical characteristics, including quantity and size, from this source can be a promising tool to identify high-risk patients [10]. However, in our previous manuscript, we did not explored the potential role of EVs cargo studied in tumor-draining pulmonary vein samples.

The EV cargo includes DNA, proteins, mRNA, and non-coding RNAs, including long non-coding RNAs (lncRNAs). lncRNAs are RNA molecules larger than 200 nt with no protein-coding capability [11]. The specificity of their expression pattern is superior to that of mRNAs and pseudogenes at tissue and disease level [12,13], making them especially interesting in the search for new biomarkers in NSCLC [14,15]. LncRNAs play key roles in the main cancer hallmarks: sustaining proliferative signaling [16], evading growth suppressors [17], activating invasion and metastasis [18], enabling replicative immortality [19], inducing angiogenesis [20], resisting cell death [21], avoiding immune destruction [22], promoting tumor inflammation [23], and deregulating cellular energetics [24]. Although lncRNAs represent only approximately 2.4% of the total exosomal RNA cargo [25], their potential as exosomal biomarkers has been studied, with promising results in other tumors [26] and in NSCLC [27]. Within exosomes, lncRNAs remain protected from RNAse-mediated degradation and their relative abundance is higher than in other extracellular vesicles [28].

In a previous study, our group characterized the prognostic role of lincRNA-p21 expression in NSCLC tumor tissue, where higher levels of lincRNA-p21 were related to shorter time to relapse (TTR) and shorter cancer-specific survival [29]. LincRNA-p21 is transcriptionally regulated by TP53 [30] and is induced under hypoxic conditions, increasing the glycolytic metabolism and malignancy of different human tumor cell lines [31]. In our previous study, we showed that the oncogenic role of lincRNA-p21 in NSCLC was associated with hypoxia and regulation of angiogenesis [29].

Considering the high tissue specificity of lncRNAs, their analysis in EVs is one of the most interesting sources of biomarkers to be explored [28]. In the present study, we have analyzed EV lincRNA-p21 expression in the tumor-draining pulmonary vein of resected NSCLC patients and correlated our findings with TTR and overall survival (OS). In addition, in NSCLC cell lines, we have explored the role of EV lincRNA-p21 in the regulation of angiogenesis and the modulation of the microRNA EV cargo.

## 2. Results

### 2.1. Patients and Extracellular Vesicles

This study was conducted in a cohort of 56 resected NSCLC patients with available blood from the tumor-draining pulmonary vein, obtained during surgery. In all samples, EVs were correctly purified. The characterization of isolated EVs had previously been performed by our group in these same samples and in others not included in the present study [10]. None of the patients received neoadjuvant treatment, but 20 (35.7%) received adjuvant chemotherapy (2 stage I, 14 stage II, and 4 stage III). Median follow-up time was 38.37 months (Interquartile range, IQR: 23.53–48.57). During follow-up, 17 patients relapsed, 13 of whom died. The main clinical characteristics of the patients are summarized in Table 1. 

Higher disease stage, adjuvant treatment, and mutated *TP53* were associated with shorter TTR (*p =* 0.016, *p* = 0.036, and *p =* 0.009, respectively) and higher disease stage and mutated *TP53* were associated with shorter OS (*p =* 0.05 and *p =* 0.015, respectively).

### 2.2. Extracellular Vesicle lincRNA-p21 from the Tumor-Draining Pulmonary Vein Impacts Prognosis in NSCLC

**Figure 1:**
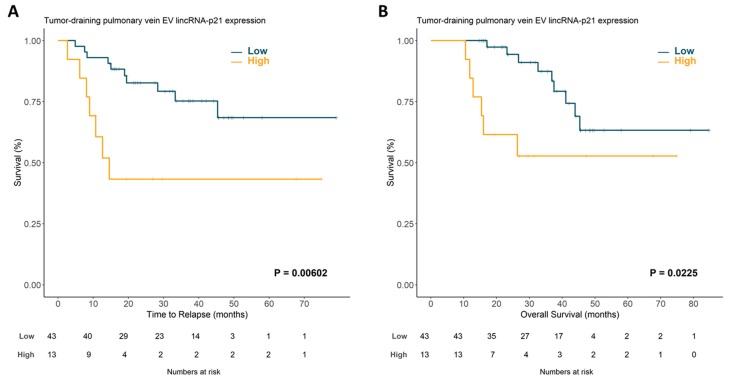
Time to relapse (**A**) and overall survival (**B**) according to lincRNA-p21 expression in tumor-draining pulmonary vein exosomal samples from non-small cell lung cancer (NSCLC) patients. EV, extracellular vesicle.

LincRNA-p21 expression was correctly determined in EVs from all patient samples. No significant associations were observed between lincRNA-p21 EV levels and the main clinic-pathological characteristics. Once the optimal cutoff for lincRNA-p21 expression was determined using X-tile [32], 13 (23.2%) patients were classified as having high expression and 43 (76.8%) as having low expression. Patients with high expression of lincRNA-p21 had shorter TTR (37.67 vs. 61.76 months; *p =* 0.00602; Figure 1A) and shorter OS (46.94 months vs. 66.83; *p =* 0.0225; Figure 1B) than those with low expression.

In order to explore the potential prognostic impact of linc-RNA-p21 expression in blood obtained from the peripheral vein, we performed the same analysis in a cohort of 45 patients with available paired peripheral vein blood. However, we found no significant difference in TTR (*p* = 0.84) or OS (*p* = 0.885) between the patients with high or low expression of lincRNA-p21 (Supplementary Figure S1).

EV lincRNA-p21 expression, disease stage, adjuvant treatment, and *TP53* mutational status were included in the multivariate analyses. High EV lincRNA-p21 expression emerged as an independent prognostic marker for shorter TTR (hazard ratio (HR): 6.129; 95% confidence interval (CI): 1.665–22.552; *p* = 0.006) and shorter OS (HR: 3.745; 95% CI: 1.113–12.604; *p* = 0.033). Disease stage and *TP53* mutational status were independent factors for TTR and OS (Table 2).

Additionally, we performed a time-dependent ROC curve estimation from censored survival data according to the high or low lincRNA-p21 expression in EVs samples. The results for the 6, 12, 24, and 36 months showed an area under the curve (AUC) of 0.639, 0.737, 0.686, and 0.632, respectively (Supplementary Figure S3).

### 2.3. Extracellular Vesicle lincRNA-p21 Expression is Induced Under Hypoxic Conditions

**Figure 2:**
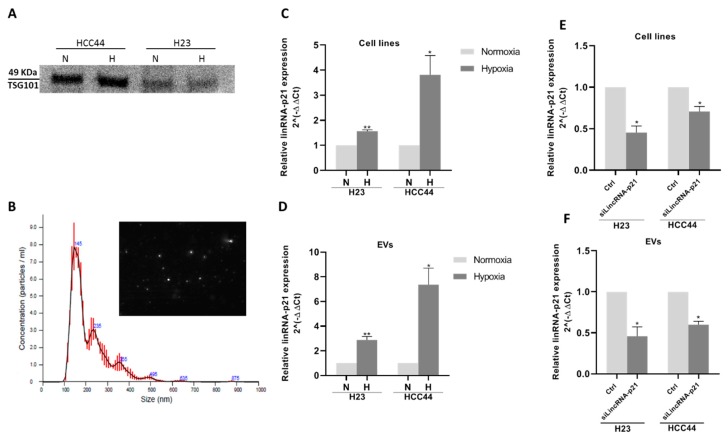
(**A**) Western blot against exosomal marker TSG101 from HCC44 and H23 cell lines under normoxic or hypoxic conditions. (**B**) Nanoparticle tracking analysis (NTA) report of the average distribution of particle size in a representative exosomal sample derived from HCC44 cells; lincRNA-p21 is overexpressed under hypoxic conditions in both (**C**) cell lines and (**D**) EVs. siRNA transfection against lincRNA-p21 under hypoxic conditions reduced its expression in both (**E**) cell lines and (**F**) EVs.

We cultured two NSCLC cell lines, H23 and HCC44, under hypoxic conditions and evaluated lincRNA-p21 levels in the cells and in their secreted EVs. Correct EV isolation from cell lines was verified using Western blot against EV marker TSG101 and by nanoparticle tracking analysis (NTA) in both normoxic and hypoxic conditions. TSG101 was detected in the purified EVs (Figure 2A) and the mode size of EVs was less than 150 nm in all samples (Figure 2B). 

LincRNA-p21 expression was upregulated in hypoxic conditions 1.6 times in H23 (*p* = 0.004) and 3.5 times in HCC44 (*p* = 0.03; Figure 2C). Interestingly, lincRNA-p21 expression was also upregulated in their secreted EVs: 2.7 times in H23 EVs (*p* = 0.005) and 7.4 times in HCC44 EVs (*p* = 0.04; Figure 2D). The hypoxia-induced upregulation was more marked in EVs than in parental cell lines. 

We also compared the expression of lincRNA-p21 in the cell lines and in their secreted EVs in both normoxic and hypoxic conditions. We found that EVs were enriched in lincRNA-p21 compared with the parental cell lines in both H23 and HCC44 in both normoxic and hypoxic conditions (Supplementary Figure S2).

### 2.4. Extracellular Vesicle lincRNA-p21 Silencing Modulates Angiogenesis and Tumor Cell Adhesion to Endothelial Cells

In order to assess if lincRNA-p21 inhibition affected EV function, we silenced cellular lincRNA-p21 expression and collected the secreted EVs. LincRNA-p21 silencing led to a significant downregulation of cellular (fold change of silenced vs. control in H23: 0.45; *p =* 0.02; HCC44: 0.70; *p =* 0.04; Figure 2E) and EV lincRNA-p21 levels in both cell lines (fold change of silenced vs. control in H23 EVs: 0.45; *p =* 0.04; HCC44 EVs: 0.59; *p =* 0.01; Figure 2F).

**Figure 3:**
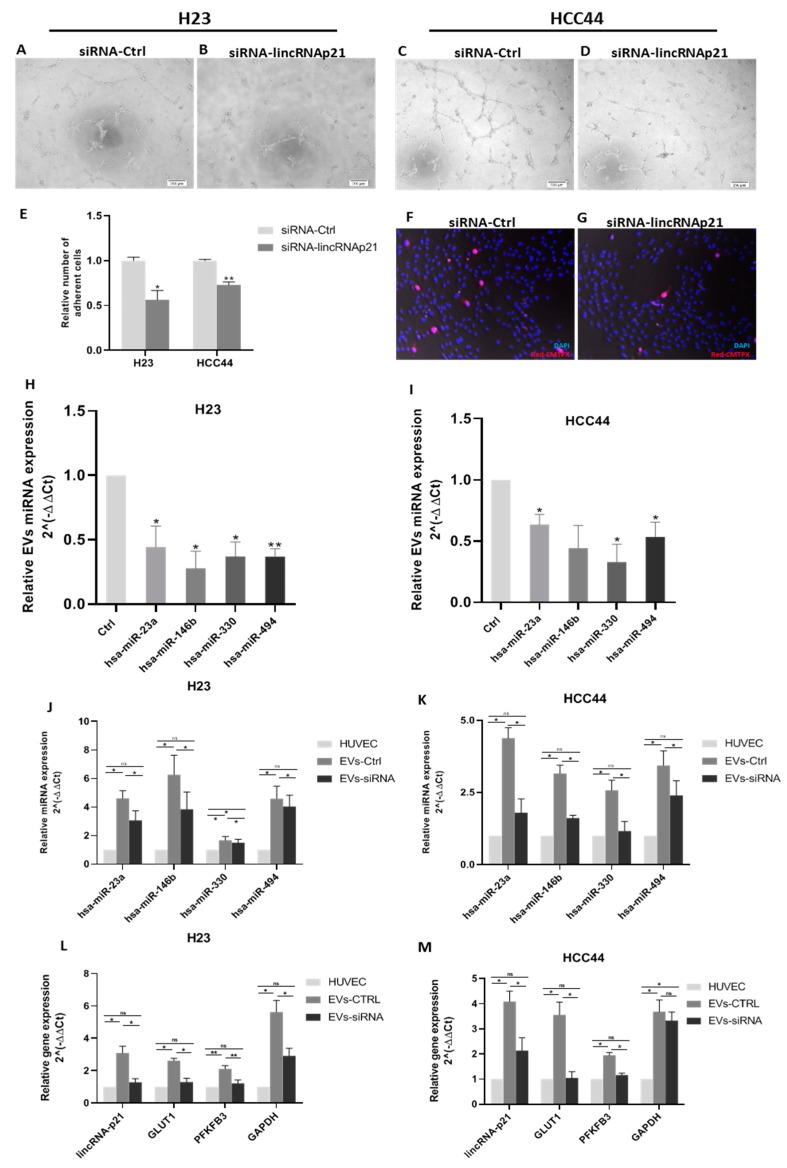
Functional in vitro assays to evaluate the modulation of angiogenesis and endothelial cell permeability by EV treatment. (**A**–**D**) Tube formation assay using human umbilical vein endothelial cells (HUVECs) treated with control EVs in H23 (**A**) and HCC44 (**C**) and lincRNA-p21-silenced EVs in H23 (**B**) and HCC44 (**D**). (**E**) Quantification of relative number of adherent cells in a cell adhesion assay using a monolayer of HUVECs treated for 12 h with control EVs or lincRNA-p21-silenced EVs. (**F**,**G**) A representative image of HCC44 cell adhesion assay. Blue staining using DAPI indicates nucleus and HCC44 cells were stained with cell tracker in red (20× magnification). (**H**,**I**) lincRNA-p21 silencing reduced the EV levels of miR-23a, miR-146b, miR-330, and miR-494 in both cell lines. (**J**,**K**) EV conditioning increases the expression of miR-23a, miR-146b, miR-330, and miR-494 in HUVECs. (**L**,**M**) EV conditioning increases the expression of lincRNA-p21, *GLUT1*, *PFKFB3*, and *GAPDH* in HUVECs. * *p* < 0.05; ** *p* < 0.01.

Next, we treated human umbilical vein endothelial cells (HUVECs) with EVs from lincRNA-p21-silenced or control H23 or HCC44 cells and performed a tube formation assay to evaluate their proangiogenic potential. LincRNA-p21 silencing impaired the tube formation capability of HUVECs in both cell lines (Figure 3A–D).

Additionally, in order to study how EVs modulate the interaction of tumor cells with endothelial cells, we performed a tumor cell adhesion assay, in which a monolayer of HUVEC cells was treated with EVs derived from lincRNA-p21-silenced or control cells prior to the addition of tumor cells to evaluate their adhesion capability. LincRNA-p21-silenced EVs reduced the capability of tumor cells to stick to HUVEC cells (H23: *p* = 0.017; HCC44: *p* = 0.001, Figure 3E), indicating a lower extravasation potential. A representative image of HCC44 is shown in Figure 3F,G.

### 2.5. LincRNA-p21 Inhibition Affects Extracellular Vesicles microRNA Cargo

We studied the expression levels of four microRNAs, selected for their bibliographic relevance in EVs or endothelial cells: miR-23a, miR-146b, miR-330, and miR-494. All four microRNAs showed a downregulation of their EV expression after lincRNA-p21 silencing in H23 (Figure 3H) and HCC44 (Figure 3I) cell lines. Fold change expression of silenced versus control in H23: miR-23a: 0.44 (*p =* 0.04); miR-146b: 0.27 (*p =* 0.01); miR-330: 0.37 (*p =* 0.01); and miR-494: 0.36 (*p =* 0.002); and in HCC44: miR-23a: 0.63 (*p =* 0.02); miR-146b: 0.44 (*p =* 0.09); miR-330: 0.33 (*p =* 0.02); and miR-494: 0.53 (*p =* 0.03).

### 2.6. Treatment of HUVECs with EVs Affects microRNA Content and Produces a Metabolic-Related Genes Activation

We evaluated the expression of the previous microRNAs in HUVECs after treatment with EVs from control or silenced lincRNA-p21 cells. After treatment, the expression of these microRNAs in HUVECs increased in comparison with basal levels (non-treated group), with a higher increment in the EV-control group (Figure 3J,K). In both cell lines and for all microRNAs analyzed, the HUVECs treated with control EVs had significantly higher levels of microRNAs than cells treated with silenced lincRNA-p21 EV (*p* < 0.05).

Additionally, we determined the expression of lincRNA-p21 after treatment with EVs to evaluate if lincRNA-p21 was also transferred to HUVECs. Our results showed that lincRNA-p21 expression increased significantly in HUVECs only after treatment with control-derived EVs, which were enriched in lincRNA-p21, in both cell lines (Figure 3L,M).

To evaluate whether the EV-mediated transfer of microRNAs and lincRNA-p21 was related to endothelial cell activation, we analyzed the levels of three previously described genes whose expression is upregulated during tumor endothelial cell activation, *GLUT1*, *PFKFB3,* and *GAPDH* [33] (Figure 3L,M). In comparison with basal levels (non-treated HUVECs), the three genes showed significantly higher levels in the HUVECs treated with control EV. This indicates that the silencing of lincRNA-p21 in the cell affects the EV content and finally reduces the endothelial cell activation.

### 2.7. Extracellular Vesicle lincRNA-p21 Expression in Patient Tumor-Draining Pulmonary Vein Correlates with EV microRNA Cargo

**Figure 4:**
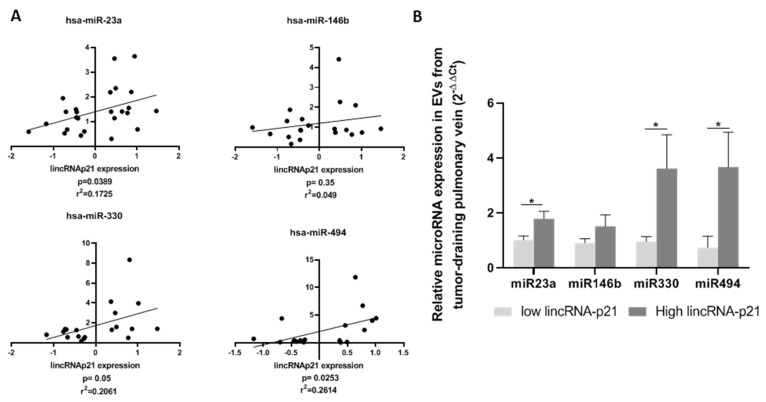
(**A**) Positive correlation between the expression of lincRNA-p21 and microRNAs miR-23a, miR-146b, miR-330, and miR-494. (**B**) EV expression levels of miR-23a, miR-146b, miR-330, and miR-494 in patient samples according to lincRNA-p21 expression levels.

We studied the expression of the previously analyzed microRNAs in EVs derived from the tumor-draining pulmonary vein of 26 patients classified according to their EV lincRNA-p21 expression. A positive correlation was observed between EV lincRNA-p21 expression and the expression of miR-23a (r^2^ = 0.175, *p =* 0.039), miR-146b (r^2^ = 0.049, *p =* 0.35), miR-330 (r^2^ = 0.206, *p =* 0.05), and miR-494 (r^2^ = 0.261, *p =* 0.025) (Figure 4A). When we classified the patients according to the prognostic cutoff in groups with low (n = 13) or high (n = 13) lincRNA-p21 expression, we observed significant differences for all microRNAs except for miR-146b. Patients with high levels of lincRNA-p21 had significantly higher levels of the microRNAs (mean expression in low EV lincRNA-p21 vs. high EV lincRNA-p21: miR-23a: 1.02 vs. 1.78, *p =* 0.0237; miR-146b: 0.91 vs. 2.73, *p =* 0.154; miR-330: 0.96 vs. 3.61, *p =* 0.045; and miR-494: 0.74 vs. 3.67, *p =* 0.034) (Figure 4B).

## 3. Discussion

To our knowledge, this study provides the first evidence of the prognostic role of EV lincRNA-p21 levels in any cancer. Although the EV expression of lincRNA-p21 has previously been identified as a diagnostic tool in prostate cancer, its prognostic relevance was not explored [34]. It has also been reported that serum lincRNA-p21 expression can be used to detect liver cell damage related to a spectrum of different diseases [35,36]. In contrast, plasma/serum levels of lincRNA-p21 failed in other studies to serve as a biomarker of treatment response in head and neck cancer or as a screening biomarker for colorectal carcinoma [37,38]. Our results indicate that EV levels of lincRNA-p21 from the tumor-draining pulmonary vein may well be an independent prognostic marker for TTR and OS in resected NSCLC patients. As a biomarker combination could be more powerful than a single biomarker, we decided to evaluate, in the same EV samples, the expression of other lncRNAs whose expression in tissue impacted prognosis in other cancers studied by our group [39,40], including HOTTIP, HOTAIR, HOXA11, and HOTAIRM1. However, for the lncRNAs HOXA11, HOTAIRM1, and HOTAIR, the expression was detected in only five (8%), two (3.5%), and zero (0%) samples, respectively. In contrast, HOTTIP was detected in all samples, but no association with TTR was observed (*p* = 0.4386). We consider that these results might highlight the relevance of the EV prognostic role of lincRNA-p21. The identification of biomarker combinations is usually derived from profiling studies, where a high number of genes are analyzed together. This is not the case of the present study, where a driven hypothesis based on previously published data led us to speculate on the potential role of exosomal lincRNA-p21 as a relapse biomarker.

LincRNA-p21 transcriptional activation has been related to *TP53* response and to HIF-1α activation under hypoxic conditions [30,31]. Our previous study in tumor tissue found no relation between lincRNA-p21 expression levels and *TP53* mutational status, but found that it was induced under hypoxic conditions in vitro [29]. In the present study, we have obtained similar results with EV lincRNA-p21 expression, with no significant correlation between lincRNA-p21 levels and *TP53* mutational status, but overexpression of EV lincRNA-p21 under hypoxic conditions. Interestingly, upregulation of lincRNA-p21 was higher in EVs than in cells in both NSCLC cell lines analyzed. Several studies have examined lincRNA-p21 in response to hypoxic conditions, relating high levels of lincRNA-p21 with a higher resistance to hypoxic stress, migration, and proliferation in vitro [41,42]; however, this is the first evidence of EV lincRNA-p21 related to hypoxia.

Importantly, lincRNA-p21 was overrepresented in EVs when compared with their parental cell lines. Gezer et al. had previously described this phenomenon [43], showing that several lncRNAs with low expression levels in cultured cells were highly enriched in derived EVs. Specifically for lincRNA-p21, there was a fold change between EVs and parental cell lines of 6500 in HeLa cells and 78 in MCF-7 cells. For gene expression calculation, Gezer et al. used a housekeeping gene to normalize and compare gene expression between cells and EVs. In our study, we have used a different approach, as the expression of housekeeping genes may differ between cells and EVs, which could introduce a bias in gene expression calculation.

We have also demonstrated that the silencing of lincRNA-p21 in cell lines impairs its EV levels. To explore the functional effect of EV lincRNA-p21 downregulation after silencing lincRNA-p21, we used two in vitro procedures: the HUVEC tube formation assay and the cell adhesion assay. We found that EVs derived from lincRNA-p21-silenced cells impaired the capability of HUVECs to form tubular structures. Tube formation assay is a standard method to assess the proangiogenic potential of soluble factors and other molecules [44]. Our results indicate that lincRNA-p21-enriched EVs promote angiogenesis. Additionally, it has been shown that tumor-derived EVs can promote cell adhesion to endothelial cells (reviewed in [45]). We explored whether tumor-derived EVs from lincRNA-p21-silenced or control cells were able to induce HUVEC preconditioning in order to enhance adhesion of tumor cells to endothelial cells. Importantly, when lincRNA-p21 expression was inhibited, the tumor-cell adhesion to endothelial cells was significantly reduced (H23: 44%; HCC44: 30%). It has previously been shown that the capacity of adhesion of tumor cells to endothelial cells is correlated with the metastatic capability of tumor cells in vitro and in vivo [33], indicating that tumor-derived EVs enriched in lincRNA-p21 promote metastasis through endothelial cell regulation.

Finally, we explored how EV cargo changes after lincRNA-p21 silencing by determining the expression levels of selected EV microRNAs previously related to angiogenesis or metastasis [45]. We studied EV miR-23a, miR-146b, miR-330, and miR-494 levels. MiR-23a has been related to epithelial–mesenchymal transition in NSCLC [46], and Hsu et al. reported that, under hypoxic conditions, EVs from NSCLC were enriched in miR-23a, which promotes angiogenesis and vascular permeability [47]. MiR-146b has been described as both an oncomiR and a tumor suppressor in NSCLC [47,48]. In hypoxic conditions, miR-146b has been related to apoptosis resistance in cardiomyocytes and to the enhancement of proliferation and migration of endothelial cells [49,50]. MiR-330 has been described as a oncogene in NSCLC and shown to promote tumor cell invasion, migration, and metastasis [51]. It has also been related to the induction of the p-STAT3/HIF-1α pathway, to increased expression of proangiogenic factors such as vascular endothelial growth factor A (VEGFA), and to the inhibition of vascular normalization [52]. Finally, miR-494 has been related to tumor angiogenesis in NSCLC [53] and is upregulated under hypoxic conditions, conferring resistance to apoptosis [54]. In the present study, lincRNA-p21 silencing in NSCLC cell lines reduced the expression of all four miRNAs in the tumor-derived EVs in both the H23 and HCC44 cell lines, indicating a reduction in the proangiogenic and tumorigenic potential of the EVs.

Additionally, we studied how this EV cargo affects the expression of these microRNAs in HUVECs after co-culture with control or lincRNA-p21-silenced EVs. All four microRNAs increased their expression in HUVECs after EV treatment, in line with the results derived from the functional studies. It has been previously reported that EV transference of miR-23a from lung cancer cells treated with radiotherapy enhances HUVEC proliferation and migration [55], and that the conditioned media derived from NSCLC cell lines over-expressing miR-330 leads to an increased tube formation, migration, and invasion capabilities of HUVECs [56]. Furthermore, we also studied the expression of lincRNA-p21, *GLUT1, PFKFB3,* and *GAPDH* in HUVECs after EV co-culture. Our results showed that all four genes significantly increased their expression after EV co-culture. Previous studies have reported that over-expression of *GLUT1, PFKFB3,* and *GAPDH* in endothelial cells is a common feature of tumor-activated endothelial cells, and the inhibition of *PFKFB3* leads to vessel normalization and reduces metastasis [33]. Less is known about the role of lincRNA-p21 in human endothelial cells, but our results indicate that it might be transferred from the tumor cells to the endothelial cells through EVs together with the microRNAs. Furthermore, miR-23a, miR-330, and miR-494 expression in patient EVs are positively correlated with the EV levels of lincRNA-p21.

We are aware that the results from the present manuscript are constrained by several limitations. For the patient’s results, the main limitation is the lack of an independent validation study. The challenging access to an independent cohort of samples from tumor-draining pulmonary vein makes the validation of the results difficult as most of the groups are working with peripheral vein and there are no available data on public repositories for in silico validation. Regarding the in vitro results, the inclusion of additional NSCLC cells lines or other endothelial cell types could reinforce conclusions. Moreover, we only explored how lincRNA-p21 affects the EV cargo of a subset of previously described microRNA related to angiogenesis, hypoxia, and endothelial cell activation. Deeper exploration using omic-techniques could improve the comprehension of the role of lincRNA-p21 in this process.

In summary, our study sheds light on the prognostic capacity of tumor-draining pulmonary vein EV lincRNA-p21 expression in NSCLC and indicates that it may well be useful as an independent prognostic marker. This study was conducted using blood from the tumor-draining pulmonary vein, which is highly enriched in tumor-derived products compared with a peripheral vein, as previously described by our group [10]. In fact, when we analyzed the impact of EV lincRNA-p21 obtained from the peripheral vein, we found no association with TTR or OS. We have shown how lincRNA-p21 alters EV cargo under hypoxic conditions, leading to a functional downregulation of angiogenesis and vascular permeability and to the expression of microRNAs and activated-endothelial cell genes in HUVECs. However, further research on the role of EV lincRNA-p21 is needed to fully understand its relationship with relapse. Future characterization of how lincRNA-p21 levels can affect other EV molecules, such as EV membrane receptors or growth factors, will enhance our understanding of the role of lincRNA-p21 in EV-mediated disease progression.

## 4. Materials and Methods 

### 4.1. Patient Samples

We included 56 stage I–IIIa NSCLC patients who underwent complete surgical resection at Hospital Clinic of Barcelona (Barcelona, Spain). Blood samples were collected in a 4 mL EDTA tube during surgery from the tumor-draining pulmonary vein before tumor resection or vessel ligation. Plasma was obtained by centrifugation (2000 rpm, 10 min) and stored at −80 until EV isolation. Approval for the study was obtained from the Clinical Research Ethics Committee of the Hospital Clinic of Barcelona (project approval number HCB/2017/1052), and written informed consent was obtained from each participant in accordance with the Declaration of Helsinki.

### 4.2. Extracellular Vesicle Isolation and Characterization

EVs from patient samples were isolated as previously described [10], using 200 μL of plasma and ultracentrifugation technique in a Sorvall MX Plus Micro-Ultracentrifuge (Thermofisher Scientific, Waltham, MA, USA) with the S140AT rotor. EVs from cell culture were isolated as previously described [57] from 12 mL of conditioned medium using a Beckman Coulter Optima l-100 XP (Beckman Coulter, Brea, CA, USA) ultracentrifuge with the 70.1 Ti rotor. After isolation, EVs were resuspended in 200 μL of DPBS for EVs’ quantification and size analysis by nanotracking analysis (NTA) or to be used in the functional assays. For Western blot analysis of EV marker TSG-101 (ab83, abcam, Cambridge, UK), EVs were resuspended in 40 μL of loading buffer mix and analyzed as previously described [57]. NTA analysis was performed using a Nanosight NS300 (Malvern panalytical, Malvern, UK) in the ICTS (Infraestructuras Científicas y Técnicas Singulares) “NanoBiosis” (Biomaterial Processing and Nanostructuring Unit of the CIBER in Bioengineering, Biomaterials, & Nanomedicine at Institut de Ciència de Materials de Barcelona, CSIC, UAB, Bellaterra, Barcelona, Spain).

### 4.3. RNA Extraction and Gene Expression Analysis

Total RNA was isolated from cell lines using TriZol® Reagent (Life Technologies, Carlsbad, CA, USA) according to the manufacturer’s protocol. For EV RNA isolation, 750 μL of Qiazol (Qiagen, Hilden, Germany) supplemented with MS2 RNA (Roche, Basel, Switzerland) as carrier were added to the EV pellet. Once resuspended, RNA extraction was completed using miRNAeasy mini kit (Qiagen) according to the manufacturer’s instructions. The High Capacity cDNA Reverse Transcription Kit® (Applied Biosystems, Foster City, CA, USA) was used to obtain cDNA using 500 ng of total RNA for cell lines and 250 ng of total RNA from EVs. For microRNAs, a total of 10 ng of RNA was used to obtain cDNA using the TaqMan MicroRNA Reverse Transcription Kit (Applied Biosystems). Relative expression was determined by real-time PCR using the 7500 Real Time PCR device (Applied Biosystems). LincRNA-p21 levels were quantified using Custom qPCR Probes (Integrated DNA Technologies, Coralville, IA, USA) using the primers and probes described by Hall et al. [58]. Taqman microRNA assays or Taqman Gene expression assays (Life Technologies) were used to quantify the expression of miR-23a-3p (000399), miR-146b-5p (474220_mat), miR-330-3p (000544), miR-494 (Tm001041), *GLUT1* (Hs00892681_m1), *PFKFB3* (Hs00998700_m1), and *GAPDH* (Hs99999905_m1). Relative quantification was calculated using 2^−ΔΔCt^. 18S (Hs99999901_s1) was used as an endogenous control for lincRNA-p21 and miR-191 (002299) for microRNAs.

### 4.4. Cell Lines

Cryopreserved samples of the lung cancer cell lines H23 and HCC44 (American Type Culture Collection and DSMZ, respectively) were received in our laboratory and passaged for less than six months. Both cell lines were cultured in RPMI 1640 (Invitrogen, Carlsbad, CA, USA) supplemented with 10% EV-depleted Fetal Bovine Serum (Invitrogen) and were grown under recommended conditions at 37 °C, 5% CO_2_, and 95% relative humidity.

### 4.5. Cell Treatment and Transfection

On day 0, 1.5 × 10^6^ cells were seeded in a 75 cm^2^ T-flask. On day 1, cells were transfected with 200 nM of a human lincRNA-p21 siRNA, as previously described by Hall et al. [58] or Smart Pool Lincode Negative Control (Dharmacon, Lafayette, CO, USA) using Lipofectamine 2000 (Invitrogen) to inhibit lincRNA-p21. On day 2, the medium was replaced with fresh medium supplemented with 10% EV-depleted FBS (Invitrogen) and cells were placed in a hypoxia chamber (Billups Rothenberg, San Diego, CA, USA), replacing all air with a gas mixture (<1% O_2_, 5% CO_2_, 94% N_2_). LincRNA-p21 siRNA inhibition was evaluated by real-time PCR at 96 h after transfection.

### 4.6. HUVEC Tube Formation Assay

HUVEC tube formation assay was performed as previously described [29]. In brief, 90 µL of a cell suspension containing 7500 HUVECs was added on top of a previously matrigel-coated well (96-well plate). Then, 10 µL of DPBS containing 250 × 10^6^ EVs derived from either lincRNA-p21-silenced cells or control cells was added. The tube formation was evaluated at 6 h, 8 h, and 12 h by phase-contrast microscopy observation using cellSense Entry 1.7 software (Olympus, Tokyo, Japan).

### 4.7. Cell Adhesion Assay

First, 4 × 10^5^ HUVECs were seeded onto an eight-well culture chamber slide (μ-Slide 8 well, IBIDI, Gräfelfing, Germany) and allowed to reach confluence. At this point, a suspension containing 800 × 10^6^ EVs derived from either lincRNA-p21-silenced or control cells was added to each well and co-cultured with HUVECs for 12 h. After incubation, a suspension containing 6000 HCC44 NSCLC tumor cells stained with the CellTracker™ Red CMTPX Dye according to manufacturer’s instructions (Life Technologies) was added to each well. Cells were co-cultured for 90 min in a cell incubator at regular conditions, after which the wells were gently washed using DPBS and fixed with paraformaldehyde 2% for 5 min. Finally, the cells were stained using DAPI and observed under an epifluorescence motorize microscope AF6000 (Leica, Wetzlar, Germany) and full-well mosaic images were obtained.

### 4.8. HUVEC Treatment with Tumor Cell-Derived EVs

First, 5 × 10^4^ HUVEC cells were seeded onto a 12-well plate and co-cultured with 4 × 10^8^ EVs derived from either control or lincRNA-p21-silenced H23 and HCC44 cell lines. After 24 h of co-culture, RNA from HUVECs was isolated and the expression of microRNAs and genes was determined.

### 4.9. Statistical Analyses

TTR was defined as the time from surgery to recurrence or last follow-up. OS was defined as the time from surgery to the date of death by any cause or last follow-up. Kaplan–Meier curves for TTR and OS were generated and compared by means of a log-rank test. All factors with *p* < 0.1 in the univariate analysis were included in the Cox multivariate regression analyses for TTR and OS. Paired or non-paired t-tests were used for comparisons between two groups. Optimal cut-offs of lincRNA-p21 expression data for TTR and OS were obtained using X-Tile program [32] and confirmed by the Kaplan–Meier test. Time-dependent ROC curves were constructed and compared using the survivalROC package of R (The comprehensive R archive network; CRAN), as previously described in [59]. All statistical analyses were performed using PASW Statistics 18 (SPSS Inc., IBM, Armonk, NY, USA) and R v2.8.1.

## 5. Conclusions

The quantification of EV lincRNA-p21 levels from the tumor-draining pulmonary vein is a potential biomarker for TTR and OS in resected NSCLC patients. Patients with high EV lincRNA-p21 have worse outcome after surgery than patients with low levels. LincRNA-p21 regulates the microRNA EV cargo and affects endothelial cell behavior, modulating angiogenesis, endothelial cell permeability, microRNA, and gene expression.

## Figures and Tables

**Table 1:** Main clinical characteristics of the 56 patients and univariate *p*-values (log-rank test) for time to relapse (TTR) and overall survival (OS).

Characteristics	Subtypes	N (%)	TTR*	OS*
**Sex**	Male	38 (67.9)		
	Female	18 (32.1)	0.802	0.953
**Age, years**	Mean age (range)	63 (32–80)		
	≤65	32 (57.1)		
	>65	24 (42.9)	0.845	0.177
**Stage**	I	29 (51.8)		
	II	21 (37.5)		
	III	6 (10.7)	**0.016**	**0.05**
**Histological subtype**	Adenocarcinoma	33 (58.9)		
	Squamous cell carcinoma	14 (25.0)		
	Other	9 (17)	0.715	0.989
**ECOG PS**	0	25 (44.6)		
	1	31 (55.4)	0.989	0.175
**Adjuvant treatment**	Yes	20 (35.7)	**0.036**	0.233
**Relapse**	Yes	17 (30.4)		
**Type of surgery**	Lobectomy/bilobectomy	44 (78.6)		
	Pneumonectomy	6 (10.7)		
	Segmentectomy	5 (8.9)		
	Atypical resection Anatomical resection	1 (1.8) 4 (7.6)	0.438 0.482	0.543 0.407
**Smoking history**	Current smoker	30 (53.5)		
	Former smoker	23 (41.1)		
	Never smoker	3 (5.4)	0.901	0.197
**Molecular features**	*KRAS* mutation	7/48 (14.9)	0.288	0.449
	*TP53* mutation	21/48 (43.8)	**0.009**	**0.015**

ECOG PS: Eastern Cooperative Oncology Group Performance Status. * Significant values are shown in bold.

**Table 2:** Multivariate analysis for time to relapse and overall survival. CI, confidence interval; EV, extracellular vesicle.

**Time to Relapse**	**Hazard Ratio (95% CI)**	***p***
Stage I	0.158 (0.037–0.668)	0.012
TP53 mutated	3.898 (1.175–12.93)	0.026
High EV lincRNA-p21	6.129 (1.665–22.552)	0.006
Adjuvant Treatment	1.771 (0.145–2.206)	0.411
**Overall Survival**	**Hazard Ratio (95% CI)**	***p***
Stage I	0.210 (0.052–0.846)	0.028
TP53 mutated	3.878 (1.053–14.282)	0.042
High EV lincRNA-p21	3.745 (1.113–12.604)	0.033

## Supplementary Materials

The following are available online at https://www.mdpi.com/2072-6694/12/3/734/s1, Figure S1: Comparison of Kaplan-Meier survival analysis between EV lincRNA-p21 expression and time to relapse (TTR) and overall survival (OS) in paired tumor-draining pulmonary vein and peripheral vein samples (n = 44). (**A**) TTR according to pulmonary vein EV lincRNA-p21 expression. (**B**) TTR according to peripheral vein EV lincRNA-p21 expression. (**C**) OS according to pulmonary vein EV lincRNA-p21 expression. (**D**) OS according to peripheral vein EV lincRNA-p21 expression. Figure S2: Comparison of lincRNA-p21 expression between cell lines and its derived exosomes. Figure S3: Time-dependent ROC curve estimation from censored survival data using survivalROC package of R and the results for the 6, 12, 24 and 36 months. LincRNA-p21 dichotomized expression according to prognostic cutoff value was used.
