# Supplementary material for: Extracellular Vesicle lincRNA-p21 Expression in Tumor-Draining Pulmonary Vein Defines Prognosis in NSCLC and Modulates Endothelial Cell Behavior
Source: Cancers (Basel). 2020 Mar 20;12(3):734. doi: 10.3390/cancers12030734 (PMC7140053; doi:10.3390/cancers12030734)

Supplementary Figure 1. Comparison of Kaplan-Meier survival analysis between EV lincRNA-p21 expression and time to relapse (TTR) and overall survival (OS) in paired tumor-draining pulmonary vein and peripheral vein samples (n=44). (**A**) TTR according to pulmonary vein EV lincRNA-p21 expression. (**B**) TTR according to peripheral vein EV lincRNA-p21 expression. (**C**) OS according to pulmonary vein EV lincRNA-p21 expression. (**D**) OS according to peripheral vein EV lincRNA-p21 expression.

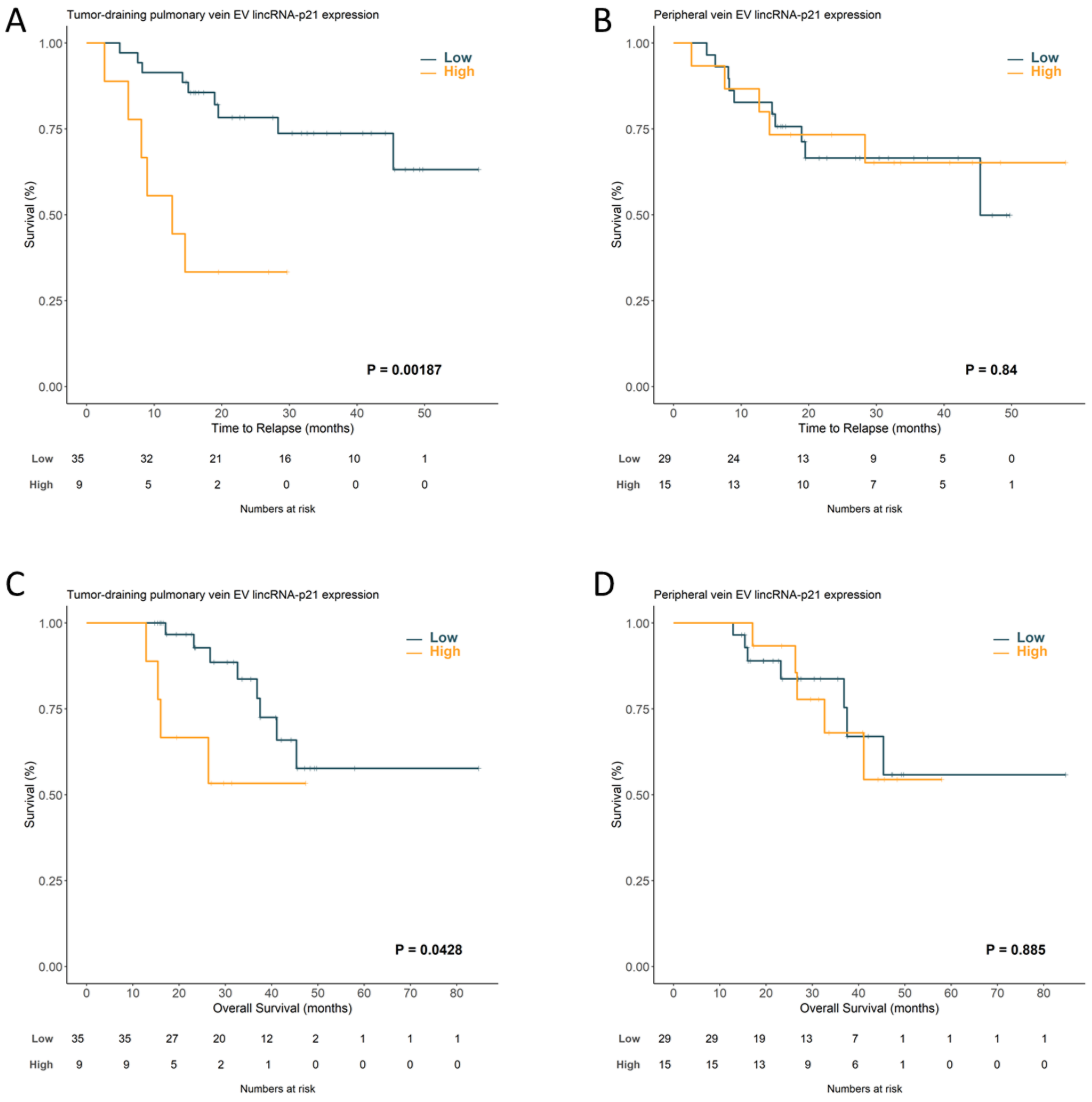

Supplement: Supplementary file 1 [file cancers-12-00734-s001.zip › Supplementary Figure 1.pdf]
