# Supplementary material for: Extracellular Vesicle lincRNA-p21 Expression in Tumor-Draining Pulmonary Vein Defines Prognosis in NSCLC and Modulates Endothelial Cell Behavior
Source: Cancers (Basel). 2020 Mar 20;12(3):734. doi: 10.3390/cancers12030734 (PMC7140053; doi:10.3390/cancers12030734)

Supplementary Figure 2: Comparison of lincRNA-p21 expression between cell lines and its derived exosomes for **A)** H23 cell line normalizing the lincRNA-p21 expression against RNA input, **B)** HCC44 cell line normalizing the lincRNA-p21 expression against RNA input, **C)** H23 cell line normalizing the lincRNA-p21 expression against 18S expression, **D)** HCC44 cell line normalizing the lincRNA-p21 expression against 18S expression.

Methods: We confirmed that RTqPCR efficiency was equal between samples derived from EVs and cell lines. Moreover, we assumed that when cycle threshold (Ct) was reached, an equal number of target copies were generated in both samples. Using the ratio between 2Ctcell/2CtEV, we calculated an estimation for the original enrichment of lincRNA-p21 in the total cDNA of EVs compared to the total cDNA of cells. We have also used an endogenous control normalization for this comparison, obtaining similar results.

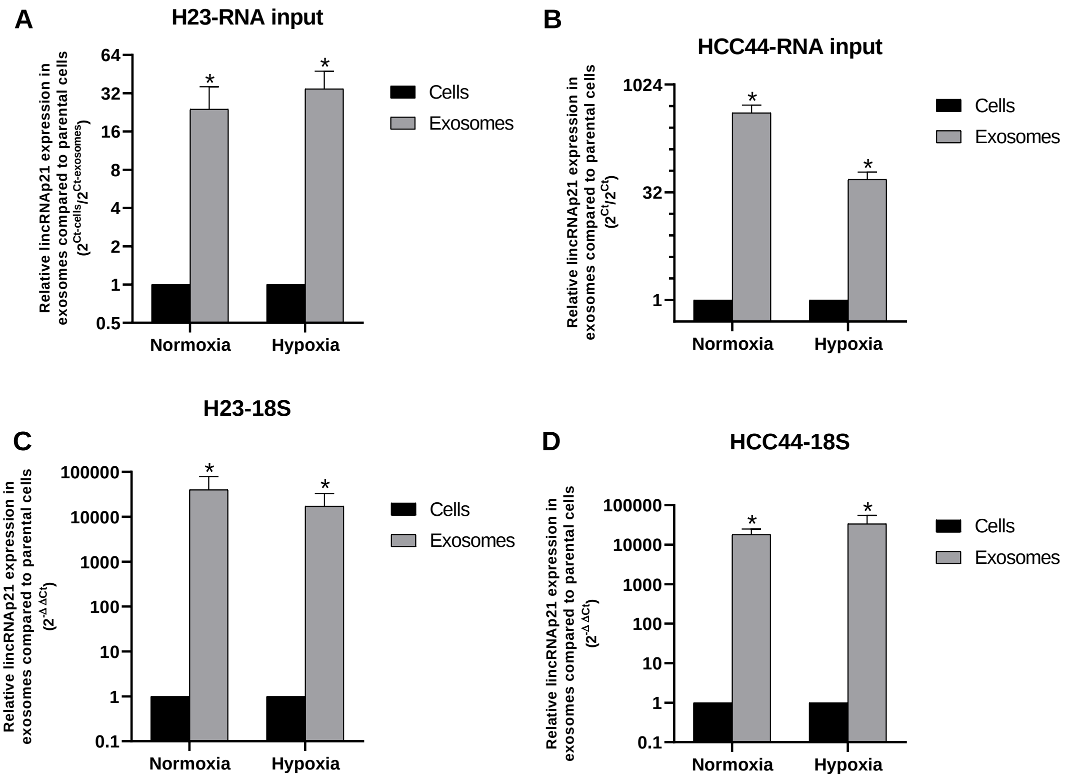

Supplement: Supplementary file 1 [file cancers-12-00734-s001.zip › Supplementary Figure 2_.pdf]
