# Supplementary material for: Extracellular Vesicle lincRNA-p21 Expression in Tumor-Draining Pulmonary Vein Defines Prognosis in NSCLC and Modulates Endothelial Cell Behavior
Source: Cancers (Basel). 2020 Mar 20;12(3):734. doi: 10.3390/cancers12030734 (PMC7140053; doi:10.3390/cancers12030734)

Supplementary Figure 3: Time-dependent ROC curve estimation from censored survival data using survivalROC package of R and the results for the 6, 12, 24 and 36 months. LincRNA-p21 dichotomized expression according to prognostic cutoff value was used.

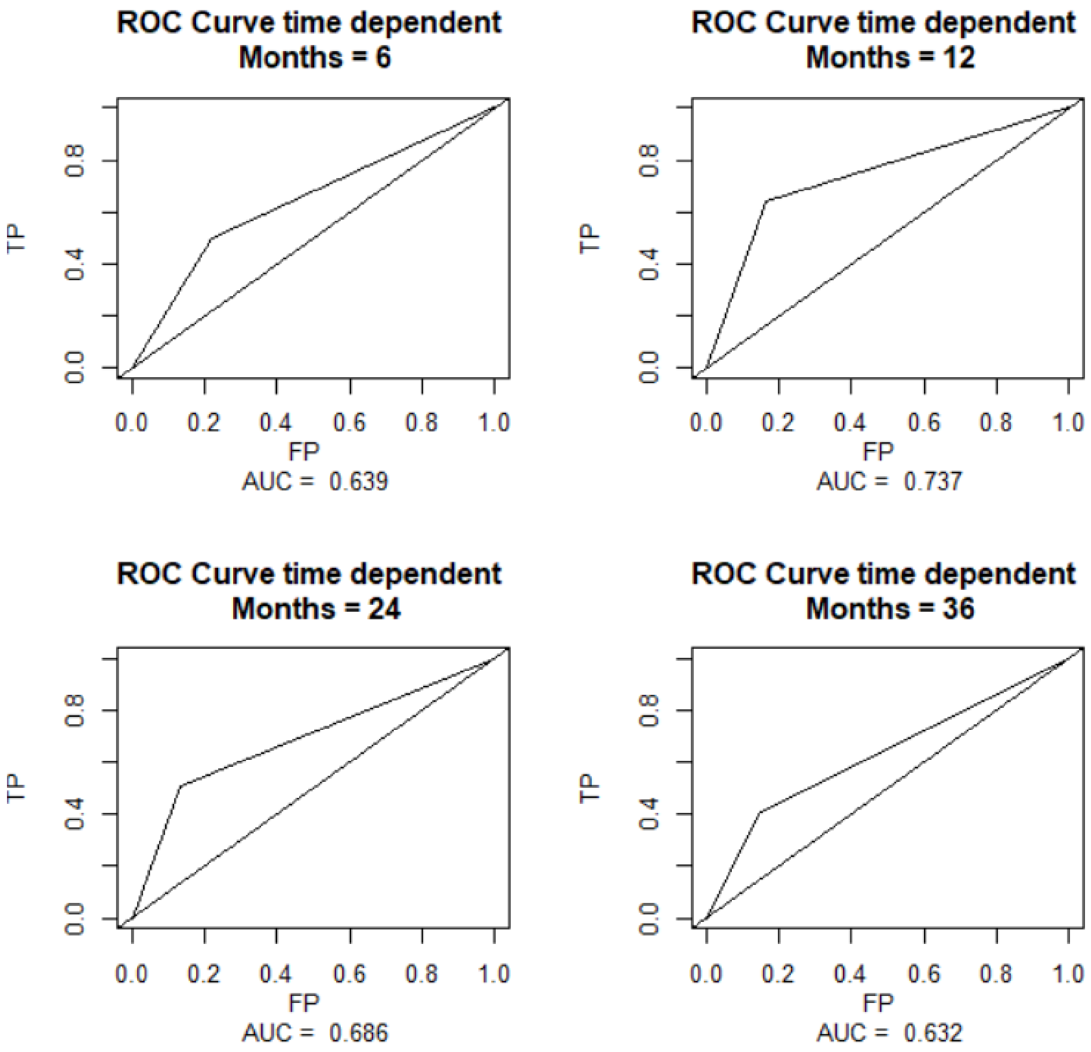

Supplement: Supplementary file 1 [file cancers-12-00734-s001.zip › Supplementary Figure 3.pdf]
